# Prevalence and Psychosocial Correlates of Mental Health Outcomes Among Chinese College Students During the Coronavirus Disease (COVID-19) Pandemic

**DOI:** 10.3389/fpsyt.2020.00803

**Published:** 2020-08-07

**Authors:** Xinli Chi, Benjamin Becker, Qian Yu, Peter Willeit, Can Jiao, Liuyue Huang, M. Mahhub Hossain, Igor Grabovac, Albert Yeung, Jingyuan Lin, Nicola Veronese, Jian Wang, Xinqi Zhou, Scott R. Doig, Xiaofeng Liu, Andre F. Carvalho, Lin Yang, Tao Xiao, Liye Zou, Paolo Fusar-Poli, Marco Solmi

**Affiliations:** ^1^ Center for Lifestyle and Mental Health, School of Psychology, Shenzhen University, Shenzhen, China; ^2^ University of Electronic Science and Technology of China, Chengdu, China; ^3^ Innsbruck Medical University, Innsbruck, Austria; ^4^ Texas A&M University, College Station, TX, United States; ^5^ Medical University of Vienna, Vienna, Austria; ^6^ Massachusetts General Hospital, Harvard Medical School, Boston, MA, United States; ^7^ Primary Care Department, Azienda ULSS 3 (Unità Locale Socio Sanitaria) "Serenissima", Dolo-Mirano District, Venice, Italy; ^8^ Anhui Jianzhu University, Hefei, China; ^9^ Arkansas State University, Jonesboro, AR, United States; ^10^ Department of Psychiatry, University of Toronto and Centre for Addiction and Mental Health (CAMH), Toronto, ON, Canada; ^11^ Alberta Health Services, Calgary, AB, Canada; ^12^ University of Calgary, Calgary, AB, Canada; ^13^ Early Psychosis: Intervention and Clinical-detection (EPIC) lab, Department of Psychosis Studies, King’s College London, London, United Kingdom; ^14^ Department of Brain and Behavioral Sciences, University of Pavia, Pavia, Italy; ^15^ Neuroscience Center, University of Padua, Padua, Italy

**Keywords:** COVID-19, pandemic, posttraumatic, anxiety, depression, university students

## Abstract

**Objectives:**

To investigate the prevalence and risk factors for poor mental health of Chinese university students during the Corona Virus Disease 2019 (COVID-19) pandemic.

**Method:**

Chinese nation-wide on-line cross-sectional survey on university students, collected between February 12^th^ and 17^th^, 2020. Primary outcome was prevalence of clinically-relevant posttraumatic stress disorder symptoms. Secondary outcomes on poor mental health included prevalence of clinically-relevant anxiety and depressive symptoms, while posttraumatic growth was considered as indicator of effective coping reaction.

**Results:**

Of 2,500 invited Chinese university students, 2,038 completed the survey. Prevalence of clinically-relevant PTSD, anxiety, and depressive symptoms, and post traumatic growth (PTG) was 30.8, 15.5, 23.3, and 66.9% respectively. Older age, knowing people who had been isolated, more ACEs, higher level of anxious attachment, and lower level of resilience all predicted primary outcome (all *p* < 0.01).

**Conclusions:**

A significant proportion of young adults exhibit clinically relevant posttraumatic stress disorder (PTSD), anxious or depressive symptoms, but a larger portion of individuals showed to effectively cope with COVID-19 pandemic. Interventions promoting resilience should be provided, even remotely, to those subjects with specific risk factors to develop poor mental health during COVID-19 or other pandemics with social isolation.

## Introduction 

Infection with the novel coronavirus (COVID-19) (1) can cause severe and potentially life-threatening pneumonia (2). The total number of confirmed cases and deaths worldwide rapidly exceeded those from the Severe Acute Respiratory Syndrome (SARS) in 2003 (3), and in March 2020 the World Health Organization declared COVID-19 a pandemic. Such results may directly affect the mental health of people (4). Although researchers worldwide race with unprecedented efforts to develop a vaccine against COVID-19, this may not be available soon enough to control the pandemic. Hence, restrictions on social contact (large-scale lock-down) and appropriate infection prevention strategies (travel limitation, quarantine, and self-isolation) have been implemented in order to prevent the spread of the virus (4).

Together with the strong person-to-person transmission capability of the virus, the strain that COVID-19 poses on public health and the implemented restrictions may have detrimental effects on mental health, including overwhelming fear and anxiety (5–7). Several individual factors can modulate mental health outcomes in reaction to such a stressful event (4). Thus, a timely evaluation of the detrimental effects of COVID-19 pandemic on mental health may inform mental health policies. In general, a group of college students is of particular interest to researchers as they experience multifaceted pressures such as academic workload, economic difficulties, and interpersonal relationship. These stressful experiences may put college students at high risk of future mental health problems (*e.g.*, depression and anxiety). For example, a meta-analytical review (including 32,694 Chinese college students) indicated the overall prevalence of depression among this age group is 23.8% (95% CI: 19.9–28.5%). Recently, a survey conducted in Eastern China found that 21.4% of 1,931 college students reported having symptoms of anxiety. Besides the two common negative emotions, posttraumatic stress disorder (PTSD) symptoms were also reported with 16.6% of 1,081 college students (8, 9). In addition, young college students typically exhibit a higher frequency of inter-individual contacts, and therefore social distancing measures (travel restrictions, quarantine, and self-isolation) during the COVID-19 pandemic may have a more profound impact on their daily life (10). Furthermore, excessive lack of or incorrect sensationalistic information by the mass media, fear of infection, witnessing death and suffering of the general population in the media, personal experiences of bereavement without the possibility to assist relatives or significant others, the impossibility to ritualize the loss with a funeral are all risk factors to develop poor mental health, beyond social isolation (11).

According to previous studies (12, 13), psychological reactions to stressful and life-threatening situations differ considerably between individuals and depend upon personal (*e.g.*, age, adverse childhood experience, attachment style, resilience) and social (*e.g.* socioeconomic status, family structure) factors. In addition to these traditional factors, the threat imminence (knowing people who had been isolated and the number of currently confirmed cases within their province in the present survey) and public understanding of health-related information approaches and prescriptions may be correlated with negative emotions during the COVID-19 pandemic. For example, during the COVID-19 pandemic, if someone they know was quarantined or infected, it may directly make them feel unsafe or insecure, ultimately resulting in negative emotions (14, 15). Therefore, the aim of the present study was to focus on young adults (university students) and to determine the prevalence of several clinically-relevant mental health outcomes (PTSD, anxiety, depression, and posttraumatic growth) and identify potential risk factors during the COVID-19 pandemic in Chinese university students. The study might help health-care providers and communities better prepare for their response to this and similar public health emergencies.

## Methods

### Study Participants

Data for the present study were collected on 12–17 February 2020, namely, around one month after the coronavirus disease (COVID-19) outbreak. Participants were recruited from more than 180 universities in China, covering a wide range of disciplines, including science, engineering, education, law, and literature. When the students participated in the survey, their locations were distributed across 29 provinces and cities of China. Prior to filling out several self-reported questionnaires, all volunteers had signed the online consent forms. In total, 2,126 students initially participated in the survey; after the exclusion of 88 individuals with missing response and incomplete responses, data on 2,038 students remained for analysis (95.9%).

### Procedure

The survey was conducted online for both convenience and safety reasons in light of the COVID-19 outbreak. Over a period of 6 days, students were invited to participate in the survey *via* Tencent’s QQ, WeChat, Weibo, and college-related websites (such as university association websites and bulletin board system forums). Participants who had completed all questionnaires that took around 15 min were given ten RMB *via* online payment (equivalent to 1.5 U.S. dollars at the current rate). Recruitment and data collection procedures were approved by the Human Research Ethics Committee (No:2020005) of Shenzhen University.

### Measurement

#### Dependent Variables

##### Zung Self-Rating Anxiety Scale (Z-SAS)

Symptoms of anxiety were assessed using the Z-SAS (16), a 20-item self-report questionnaire. The Z-SAS includes measures of state and trait anxiety based on scoring in four groups of manifestations: cognitive, autonomic, motor, and central nervous system symptoms. Responses to each item range from 1 (a little of the time) to 4 (most of the time) with higher scores indicating increased levels of anxiety. Standard scores above 50 (including 50) as a cut-off point suggest clinically significant anxiety (17, 18).

##### Patient Health Questionnaire (PHQ-9)

PHQ-9 was adopted to assess participants’ symptoms of depression during the last 7 days. The PHQ-9 uses the DSM-IV diagnostic criteria to assess depressive symptomatology (*i.e*., sleep, concentration, and energy problems, low self-esteem, anhedonia, *etc.*) on a 4-point scale ranging from 0 (not at all) to 3 (nearly every day) (19, 20). Individuals with the PHQ-9 scores of 10 or above were classified as having depressive symptoms (21, 22). In addition to its utility as a short screener, the PHQ-9 also captures depression severity. Overall scale scores are computed as a sum of the nine items, with the higher scores indicating the higher level of depressive symptoms.

##### The Abbreviated PTSD Checklist (PCL)

PTSD symptoms were assessed using the abbreviated PTSD Checklist (PCL), which has six items corresponding to each symptom (23). Responses to each item on the PCL-C were recorded on a 5-point Likert scale ranging from 1 (not at all) to 5 (extremely) indicating the degree to which a respondent had experienced a particular symptom over the previous month. The sum of the response scores was calculated, with higher scores indicating a higher level of PTSD symptoms. A cut-off score of 14 and over was used to define having PTSD symptoms.

##### The PostTraumatic Growth Inventory (PTGI)

The PIGI was utilized to assess positive self-reported experience of individuals who have experienced traumatic events (24). When the original version was adapted to be used in China, psychometric properties were good (25–27), with the internal consistency reliability of 0.95 for the culture-specific inventory and of 0.81–0.88 for four dimensions. The PIGI consists of 21 items across five dimensions, including relationship to others (seven items), new possibilities (five items), personal strength (four items), spiritual change (two items), and appreciation of life (three items). Two previous studies (26, 27) had indicated that the dimensions on religious beliefs and spiritual change do not fit into the Chinese culture. Therefore, we excluded these two items from the questionnaire we used in our study. Participants responded on a 6-point scale ranging from 0 (no change) to 5 (complete change) and could obtain a total score ranging from zero to 95, with higher scores representing higher levels of posttraumatic growth (PTG). Participants who scored above 57 (include 57) were considered to have posttrauma growth (PTG) symptoms, as recommended by a previous research (27).

#### Independent Variables

##### Sociodemographic Correlates

Participants were invited to report on their age, gender (0 = male; 1 = female), family structure (0 = an intact family (1); 1 = non-intact family), their current location (1 = Wuhan city; 2 = Hubei province except for Wuhan; 3 = Other areas except for Hubei province), Further, in order to examine whether risk level response to the COVID-19 outbreak was related to individuals’ mental health status, information on whether they knew people who had been isolated for this outbreak (0 = yes; 1 = no) and the number of confirmed cases of COVID-19 pneumonia (range from 56,249 to 48) on the days the respondents completed the questionnaires was obtained from the National Health Commission. Above 1,000 (included 1,000) confirmed patients were coded as 1 and below 1,000 were coded as 0.

##### The Adverse Childhood Experiences (ACEs) Questionnaire

The ACE was used to assess the adverse experiences of participants in the first 18 years of life (28). It contained 29 items within ten aspects: emotional abuse (two items; any respond is often or very frequent, coded as 1), physical abuse (two items; if the answer of the first item is often or very frequent, or the answer of the second item is sometimes, often, or very frequent, coded as 1), sexual abuse (four items; either answer is yes, coded as 1), emotional neglect (five items; if the total score of these five questions is >=15, coded as 1), physical neglect (five items; if the total score of these five questions is >=10, coded as 1), domestic violence (four items; either answer is sometimes, often or very frequent of the first two relevant questions, or the answer is one or two times, sometimes, often, very often in the last two items, coded as 1), household substance abuse (two items; either answer is yes, coded as 1), mental illness in the household (three items; either answer is yes, coded as 1), parental separation/divorce (one item; if the answer is yes, coded as 1), and criminal household member (one item; if the answer is yes, coded as 1). A total score of ACEs questionnaire summarized 10 dimension score and ranged from 0 to 10, with higher scores representing higher levels.

##### The Adult Attachment Scale (AAS)

The 18-item AAS was used to measure adult attachment (29, 30). In this scale, there are three attachment dimensions that are associated with anxiety and avoidance. Specifically, anxiety refers to a person who is worried about being abandoned, unloved, and rejected. Avoidance involves two conditions: (1) an individual is comfortable with closeness and intimacy; (2) an individual feels that people can be relied on to be available when needed. All items were responded using 5-point scales (1 = “not at all characteristic of me” and 5 = “very characteristic of me”). After reversing the related items, a total score of anxious attachment and avoidant attachment subscale ranged from 6 to 30, and 12–60, respectively, with higher scores indicating higher levels of insecure attachment.

##### The Connor–Davidson Resilience Scale (CD-RISC)

Resilience was assessed using the Connor–Davidson Resilience Scale, which reflects the ability to tolerate experiences related to change, personal problems, illness, pressure, failure, and painful perception (31). The original version was adapted by Chinese scholars, with good reliability and validity that were reported (32, 33). This adapted scale consisted of 10 items in which participants needed to respond using a 5-point Likert scale (0 = not true at all and 4 = true nearly all the time). The final scores can be obtained by adding up the responses (value) of all items ranging from 10 to 50, with higher total scores indicating higher resilience capacity.

##### The Subjective Socioeconomic Status (SES) Scale

The widely used subjective SES scale was adopted to assess the subjective social status of participants (34). Participants were given a drawing of a ladder with 10 rungs that was described as follows: “Think of this ladder as representing where people stand in our society. At the top of the ladder are the people who are the best off, those who have the most money, most education, and the best jobs. At the bottom are the people who are the worst off, those who have the least money, least education, and worst jobs or no job.” They were then asked to place an *X* on the rung that best represents where they think their families stand on the ladder.

### Statistical Analyses

Firstly, we described the data (predictors and primary and secondary outcomes) using means and standard deviations (SD) for continuous variables and proportions for categorical variables. We then tested the internal reliability of the survey using Cronbach’s alpha across the Adverse Childhood Experiences questionnaire, The Adult Attachment Scale with anxious and avoidant attachment subscale, The Connor–Davidson Resilience Scale, abbreviated PTSD Checklist (PCL), the Zung Self-Rating Anxiety Scale, the 9-item Patient Health Questionnaire, and The Post Traumatic Growth Inventory. Secondly, we reported the proportion of clinically relevant outcomes employing current recommendations for cut-off score for detecting clinically relevant levels of PTSD, anxiety, depression, and PTG (14, 50, 10, and 57, respectively) (23, 35–37). Thirdly, we explored the association between predictors and primary and secondary outcomes, using RIDGE regression (38). In RIDGE regression a penalizing factor is employed to reduce model complexity and prevent over-fitting which may result from simple linear regression. The penalty is equivalent to the square of the magnitude of the coefficients. Using RIDGE regression, it is possible to have an idea of the importance of predictors by sorting the absolute values of coefficients (the most important predictor has the highest absolute value of coefficients). Among the predictors we considered were: age, sex, family structure, SES, knowing people who had been isolated, current location, and the number of confirmed cases of COVID-19 pneumonia in each local area, ACEs, attachment type, resilience. All analyses were performed using the Statistical Package for the Social Sciences (SPSS, version 23.0) with p < 0.05 being set as a statistical significance (two-tailed).

## Results

### Characteristics of Participants

Participants’ information is detailed in **Table 1**, including levels of ACEs (1.30 ± 1.72), anxious attachment (17.05 ± 5.10) and avoidant attachment (31.75 ± 6.00), and resilience (35.45 ± 6.62). Reliabilities of ACEs, anxious attachment and avoidant attachment subscale, and resilience were 0.73, 0.86, 0.73, and 0.92, respectively. The mean age of participants was 20.6 (SD 1.90), and sixty-three percent of the included participants were female. Of the respondents, 91.2% were from intact families and the mean score of the subjective SES was 4.85 (SD 1.37). Regarding COVID-19 data, 12.1% indicated knowing people (family members, relatives and/or friends) who had been isolated, and on the day of data collection, 58.2% respondents were located in areas with >1,000 confirmed cases.

**Table 1:** Characteristics of participants (N = 2,038).

	Mean/N	SD/%	Cronbach’s a
**Age**	20.56	1.90	
**Sex**			
*Male*	755	37.0	
*Female*	1,283	63.0	
**Family intactness**			
*Intactness*	1,859	91.2	
*Non-intactness*	179	8.8	
**Subjective SES**	4.85	1.37	
**Knowing people who have been isolated for this outbreak**			
*Yes*	247	12.1	
*No*	1,791	87.9	
**Number of confirmed patients in participants’ area**			
*>=1000*	1,187	58.2	
*<1000*	851	41.8	
**ACEs**	1.30	1.72	0.73
**Anxious attachment**	17.05	5.10	0.86
**Avoidant attachment**	31.75	6.00	0.73
**Resilience**	35.45	6.62	0.92

Sex: 0 = male; 1 = female; Family intactness: 0 = Intactness; 1 = Non-intactness; Knowing people who have been isolated for this outbreak: 0 = yes; 1 = no; Number of confirmed patients in participants’ area: 0 < 1,000; 1 >= 1,000; Subjective SES, Subjective Socioeconomic status; ACEs, Adverse Child Experiences; Anxious attachment and Avoidant attachment, the two dimensions of Adult Attachment Scale.

### Prevalence of Clinically-Relevant PTSD, Anxiety and Depressive Symptoms, and PTG

**Figure 1:**
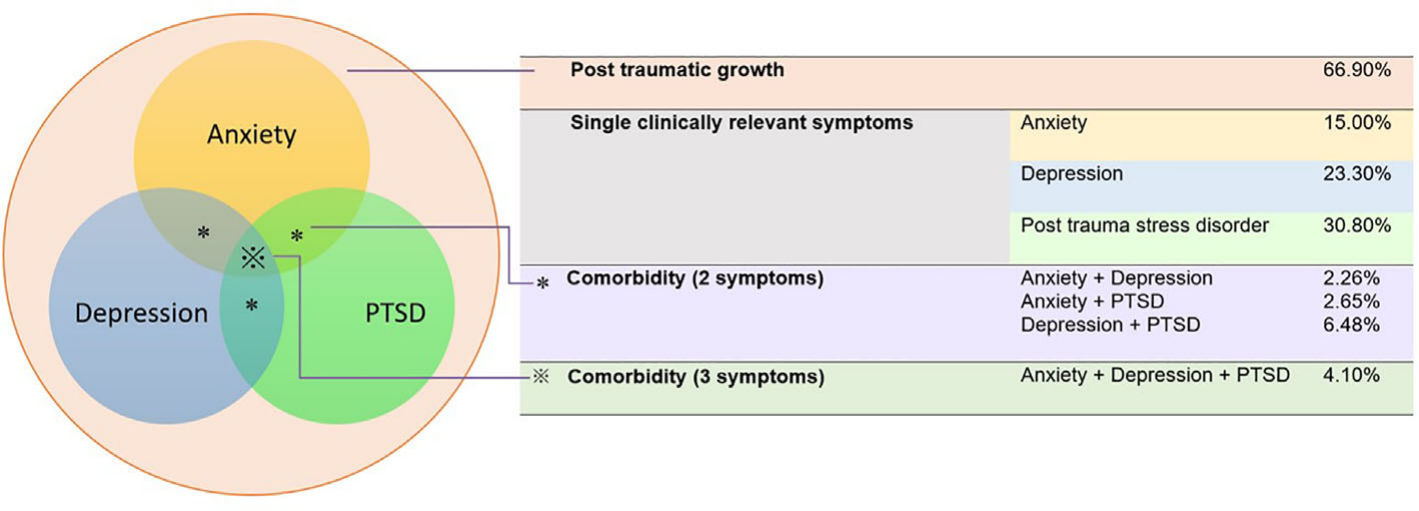
Prevalence of clinically relevant posttraumatic stress disorder (PTSD), anxiety and depressive symptoms and levels of post traumatic growth (PTG).

Severity of PTSD symptoms (11.79 ± 4.25), anxiety symptoms (40.88 ± 8.48), depressive symptoms (6.37± 4.85), and levels of PTG (60.99 ± 16.96) is presented in **Figure 1**. Cronbach alphas reflected a good internal reliability of PTSD, anxiety, depression, and PTG domain with.81,.82,.86, and.95, respectively. With respect to prevalence of clinically-relevant symptoms, 30.8% (95%CI: 28.8 to 32.8%) of the individuals completing the survey presented clinically relevant PTSD symptoms, 15.5% (95%CI: 13.5 to 16.6%) anxiety, 23.3% (95%CI: 21.5 to 25.1%) depressive symptoms, and 66.9% (95%CI: 65.1 to 69.2%) of the subjects were considered to have PTG. In addition, a significant proportion presented co-existing clinically-relevant symptoms: 4.1% (95%CI: 3.2 to 4.9%) displayed symptoms of PTSD, depression and anxiety simultaneously and 7.0% (95%CI: 6.0 to 8.2%) respectively presented symptoms of two conditions simultaneously (**Figure 1**), suggesting a highly vulnerable subgroup of individuals who develop multiple co-morbid symptoms.

### Correlates of Primary Outcome (PTSD Symptoms)

Results indicated that the regression model was significant (p < 0.001), with 10 independent variables together explaining about 13% of variance in PTSD symptoms. Specifically, older age (*β* = 0.02, p < 0.001), knowing people who had been isolated (*β* = −0.09, p < 0.001) were found to significantly predict a higher likelihood of reporting PTSD symptoms; more ACEs (*β* = 0.06, p = 0.005), higher level of anxious attachment (*β* = .18, p < 0.001), and lower level of resilience (*β* = −0.21, p < 0.001) were significantly associated with a higher probability of reporting PTSD symptoms (**Table 2**).

**Table 2:** RIDGE regression of predictors on the primary outcome (PTSD).

Predictors	PTSD			
	B	*β*	t	ΔR2
				0.130***
Age	.18	.08	4.29***	
Sex	−.004	−.0004	−.02	
Family intactness	.19	.01	.47	
Subjective SES	.09	.03	1.47	
Knowing people who have been isolated for this outbreak?	−1.17	−.09	−4.78***	
Number of confirmed patients in participants’ area	.27	.03	1.69	
ACEs	.14	.06	2.77**	
Anxious attachment	.15	.18	8.29***	
Avoidant attachment	−.01	−.02	−.86	
Resilience	−.13	−.21	−10.31***	

PTSD, posttraumatic stress disorder; *p < .05, **p < .01, ***p < .001.

### Correlates of Secondary Outcomes (Anxious and Depressive Symptoms and PTG Levels)

Firstly, the results found that the regression model was significant (p < 0.001), with 10 independent variables together explaining about 29.5% of variance in anxiety symptoms. Specifically, knowing people who had been isolated (*β* = −0.04, p = 0.008), more ACEs (*β* = 0.15, p < 0.001), higher level of anxious attachment (*β* = 0.17, p < 0.001) and avoidant attachment (*β* = 0.05, p = 0.005), and lower level of resilience (*β* =−0.34, p < 0.001) were significantly associated with greater probability of developing anxiety symptoms. Secondly, the results indicated that the regression model was significant (p < 0.001), with 10 independent variables together explaining about 21.7% of variance in depressive symptoms. Specifically, older age (*β* = 0.04, p = 0.03), knowing people who had been isolated (*β* = −0.06, p < 0.001), more ACEs (*β* = 0.12, p < 0.001), higher level of anxious attachment (*β* = 0.24, p < 0.001), and lower level of resilience (*β* = −0.22, p < 0.001) were significantly associated with greater probability of reporting depressive symptoms. Thirdly, the results indicated that the regression model was also significant (p < 0.001), with 10 independent variables together explaining about 18.3% of variance in PTG level. Specifically, when analyzing predictors of PTG levels, higher subjective SES (*β* = 0.09, p < 0.001), knowing people who had been isolated (*β* = −0.04, p = 0.03), and the number of confirmed cases in participants’ areas (*β* = −3.99, p < 0.001), fewer ACEs (*β* = −0.07, p = 0.002), lower level of avoidant attachment (*β* = −0.20, p < 0.001), and higher level of resilience (*β* = 0.23, p < 0.001) were significantly associated with the higher level of PTG. Such detailed results are presented in **Table 3**.

**Table 3:** RIDGE regression of predictors on the secondary outcomes (Anxiety symptoms, Depressive symptoms, PTG).

Independent variables	Anxiety symptoms				Depressive symptoms				PTG			
	B	*β*	T	ΔR^2^	B	*β*	t	ΔR^2^	B	*β*	t	ΔR^2^
				0.295^***^				0.217^***^				0.183***
Age	.13	.00	.05		.10	.04	2.16^*^		.25	.03	1.51	
Sex	−.06	.03	1.71		.10	.01	.58		−.76	−.02	−1.17	
Family intactness	−.30	−	−		−.22	−.01	−.68		.80	1.13	.71	
Subjective SES	−.19	−.03	−1.80		−.02	−.01	−.31		1.05	.09	4.62***	
Knowing people who have been isolated for this outbreak?	−1.16	−.04	−2.63^**^		−.94	−.06	−3.56^***^		−2.01	−.04	−2.12*	
Number of confirmed patients in participants’ area	.05	.003	.16		.29	.03	1.60		−2.55	−.07	−3.99***	
ACEs	.73	.15	8.09^***^		.33	.12	6.09^***^		−.65	−.07	−3.35***	
Anxious attachment	.28	.17	8.54^***^		.23	.24	11.53^***^		.04	.01	.64	
Avoidant attachment	.08	.05	2.83^**^		.02	.02	1.03		−.58	−.20	−9.80***	
Resilience	−.43	−.34	−18.83^***^		−.16	−.22	−11.84^***^		.59	.23	12.00***	

PTG, posttraumatic growth; *p < .05, **p < .01, ***p < .0.

## Discussion

The present study showed high prevalence rates of PTSD, anxiety and depressive symptoms as well as PTG among Chinese university students during the COVID-19 pandemic. It also identified specific risk factors for poor mental health outcomes during stressful COVID-19 pandemic. Prevalence rates of PTSD and PTG symptoms (30.8 or 66.9%, respectively) were comparable with those observed after the Sichuan earthquake which caused 69,227 deaths and 374,643 injuries in 2008 (26, 27). On one hand, college students have traumatic experiences and present some negative mental health effects during the COVID-19 pandemic. On the other hand, personal growth reflects that individuals have intrinsic motivation for positive growth and will seek to implement effective ways to cope with trauma and adversity (39, 40).

Rates of anxiety symptoms (15%) was lower than those in a similar study conducted by Chang et al. that focused on 3,881 college students who were specifically selected from universities located in Guangdong province (26.6%) (9). The higher percentage in that study may be due to the use of a different instrument (the 7-item Generalized Anxiety Disorder) (12) with different cut-offs or the fact that it was conducted earlier (between January 31, 2019 and February 3, 2020). The study was conducted one week after confirming person-to-person transmission of the COVID-19, which has increased anxiety.

Rates of depressive symptoms (23.3%) matched those (21.16%) estimated in a previous study (12). Also, two concomitant symptomatic dimensions among anxiety, depressive, and posttraumatic symptoms affected 4.1% of participants and two of them in 7.0%, confirming previous findings outside of COVID-19 context (41). Such comorbidity (*e.g.*, depression, anxiety, and PTSD) may lead to increased risk of developing chronic mental disorder relative to depression or anxiety presence alone (42), and so this unique group needs special attention.

A number of socio-demographic risk factors identified subjects with poor mental health. Higher age might be associated with more PTSD and depressive symptoms. Individually, the positive association between age and PTSD symptoms may be attributed to the fact that older students are about to experience job search competition while working on their thesis as a part of the requirements in order to successfully obtain their undergraduate degrees in China’s higher education. This result can also be partially explained by the fact that some of the juniors and seniors may be preparing for the graduate school entrance examination. The aforementioned behavior, to a large extent, causes more stress relative to those freshmen and sophomores, which may ultimately lead to higher level of PTSD and depressive symptoms during the COVID-19 pandemic (12). Moreover, higher subjective SES may predict a higher level of PTG, which is strongly supported by previous studies (43–45). Such results may arise from students with higher SES being more likely to get access to safe settings, to afford masks and health equipment, and to receive food support, which may help them effectively cope with stressful events like the COVID-19 pandemic. Knowing people who had been isolated during the COVID-19 pandemic was significantly associated with higher levels of anxiety, depression, and PTSD. It may be due to the fact that knowing someone close had been quarantined might have increased the sensation of being vulnerable. When someone we know is isolated, we become more empathetic so that naturally we think of ourselves, and then it is easy to become tense, or if the isolated person has been in contact with us before, we are even more worried about whether we will be infected. This kind of pandemic fear may cause more negative psychological feelings such as anxiety and PTSD symptoms (15, 46). Finally, participants living in areas with a higher number of confirmed cases reported lower levels of PTG, which can be attributed to that the greater number of confirmed cases makes them feel having greater possibility to be infected as they are exposed to more dangerous area. Timely mental health services should be offered in those areas with a higher number of affected people (46, 47). Surprisingly, the number of participant-based confirmed cases was not significantly predictive of negative emotions in this study. Since our survey was conducted in mid to late February which was one and half month after the initially identified outbreak, college students had a sufficient amount of time to adapt to such stressful environment and learn how to effectively manage their negative emotion as usual, or their negative emotion has reached a plateau.

Considering psychological factors, students who reported more ACEs, higher levels of insecure attachment, especially anxious attachment, and lower levels of resilience reported significantly higher level of anxiety, depression, and PTSD, whereas fewer ACEs, a lower level of avoidant attachment, and a higher level of resilience significantly predicted an increased likelihood of achieving PTG. These results replicate findings from several previous studies (48–52). Specifically, overarching biopsychosocial psychopathology models emphasize that negative life events, particularly those occurring during sensitive developmental periods such as adverse childhood experiences, play a major etiologic role in precipitating and/or exacerbating psychological disorder in later life, and that attachment determines adult internal and external thought and behavior schemata (53, 54). Individuals who score high on the Attachment–Anxiety dimension tend to intensify negative emotional states (hyper-activation strategies), whereas those with high scores on the Attachment–Avoidant dimension tend to distance themselves from emotional situations (deactivation strategies), avoid developing closes relationship, and consequently may be less sensitive to stress (53). In addition, resilience was observed to be a strong protective factor against mental health problems and promoted PTG, which is supported by previous research (48). Resilience, reflecting problem-solving ability or positive adaptation, is an individual’s ability to overcome adversities with positive emotions. It may enhance individuals’ understanding of happiness and promote psychological health (48). It indicates that under the current situation individuals with high resilience presented a “mental immunity,” which allows them to resist the psychosocial influences of major events (55). Resilience should be promoted in university students as it can reduce their psychiatric symptoms and improve their PTG under stressful situations. It is essential to translate these findings into clinical practice and mental health policy-making. Under the current circumstances online interventions targeting vulnerable populations promoting resilience might be a viable option. More specifically, resilience can be promoted in different ways such as providing a supportive social network (56), enhancing active coping self-efficacy strategies (*e.g.*, enhancing the perception that one is able to manage or recover from a stressful event) (57), learning mindfulness skills (*e.g.*, deep breath and focusing on the present moment) (58), as well as nurturing a sense of purpose of life and the ability to find meaning in face of COVID-19 pandemic (59).

The findings of the present study need to be considered in the context of several limitations. First, a cross-sectional design and corresponding measures before COVID-19 outbreak were not available, thus limiting any causal interpretation. Extending this cross-sectional survey to a longitudinal study would help to determine the developmental trajectory of mental health outcomes and predictors throughout the COVID-19 epidemic. Second, our participants were mainly from Guangdong, Anhui hui, Hebei, and Jiangsu provinces, and the most strongly affected province (Hubei) was very poorly represented (1.6%); thus, the findings may not be generalized to the Hubei province. From a practical perspective, it is particularly difficult to obtain data from college students who were most severely affected or whose family members and relatives are under quarantine/self-isolated or being diagnosed with the COVID-19. Future studies may consider data collection in Hubei province after people recovered from the COVID-19. Third, data on previous histories of mental disorders or treatment were not collected in this study, so it largely remains unknown whether thesenbsp;symptoms are associated with the COVID-19 outbreak or whether they pre-existed. Future studies may include respondents’ previous histories of mental disorders or treatment. Finally, self-report instruments were used to detect and screen anxious, depressive and PTSD symptoms, which may be considered less accurate than the rating of a clinical psychologist or psychiatrist. Future studies on this similar topic that additionally includes diagnostic instruments administered by clinical psychologists or psychiatrists are encouraged.

## Conclusions

In conclusion, this study suggests a high prevalence of posttraumatic, anxiety and depressive symptoms, but also that many have an intrinsic motivation for positive growth during the COVID-19 pandemic. These findings may help provide important information for future tracking or intervention research in this group. Specific risk factors exist to identify subjects at risk of poor mental health. While early traumatic events in childhood and insecure attachment are risk factors, resilience is a modifiable factor and a potential therapeutic target to prevent poor mental health outcome during COVID-19 pandemic.

## Data Availability Statement

The raw data supporting the conclusions of this article will be made available by the authors, without undue reservation.

## Ethics Statement

Recruitment and data collection procedures were approved by the Human Research Ethics Committee (No.2020005) of Shenzhen University. Informed consent was obtained *via* online consent forms and participants received monetary compensation (10 RMB equivalent to 1.5 U.S. dollars at the current rate).

## Author Contributions

XC and LZ had full access to all of the data in the study and took responsibility for the integrity of the data and the accuracy of the data analysis. XC and LZ contributed with concept and design. XC, LH, JW, QY, JL, XL, IG, PT, PF-P, and MS contributed with acquisition, analysis, or interpretation of data. XC and LZ contributed with drafting of the manuscript. BB, PW, MH, IG, AB, SD, AC, NV, LY, and MS contributed with critical revision of the manuscript for important intellectual content. XC and TX contributed with statistical analysis. XC contributed with administrative, technical, or material support. LZ contributed with supervision.

## Funding

This study was supported by National Social Science Foundation (No. 16CSH049) and National Natural Science Foundation (No. 31871115).

## Conflict of Interest

The authors declare that the research was conducted in the absence of any commercial or financial relationships that could be construed as a potential conflict of interest.
